# Complete Genome Sequence of *Pseudomonas balearica* DSM 6083^T^

**DOI:** 10.1128/genomeA.00217-16

**Published:** 2016-04-21

**Authors:** Antoni Bennasar-Figueras, Francisco Salvà-Serra, Daniel Jaén-Luchoro, Carolina Seguí, Francisco Aliaga, Antonio Busquets, Margarita Gomila, Edward R. B. Moore, Jorge Lalucat

**Affiliations:** a Microbiologia, Departament de Biologia, Universitat de les Illes Balears, Palma de Mallorca, Illes Balears, Spain; b Culture Collection University of Gothenburg (CCUG) and Department of Infectious Diseases, Sahlgrenska Academy, University of Gothenburg, Gothenburg, Sweden; c Clínica Rotger, Microbiologia, Palma de Mallorca, Illes Balears, Spain

## Abstract

The whole-genome sequence of *Pseudomonas balearica* SP1402 (DSM 6083^T^) has been completed and annotated. It was isolated as a naphthalene degrader from water of a lagooning wastewater treatment plant. *P. balearica* strains tolerate up to 8.5% NaCl and are considered true marine denitrifiers.

## GENOME ANNOUNCEMENT

The complete genome sequence of *Pseudomonas balearica* DSM 6083^T^ (= CCUG 44595^T^ = SP1402^T^) has been determined. This strain was originally isolated from the water of a lagooning wastewater treatment plant by enrichment with 2-methylnaphthalene at 40°C (1) and classified as a new denitrifying species of *Pseudomonas* (2). Its physiological tolerance to 8.5% NaCl suggested that *P. balearica* represents a true marine *Pseudomonas* species; other strains of *P. balearica* have been isolated from marine samples (1) and salt marshes (3).

Pure cultures of *P. balearica* DSM 6083^T^ were confirmed on Columbia agar with 5% sheep blood after incubation at 30°C for 18 to 20 h. Genomic DNA was obtained from cells at the exponential phase of growth and confirmed to be *P. balearica* by housekeeping gene sequence analyses (4). A hybrid assembly strategy, with CABOG assembler, version 8.1 (5), was applied for *de novo* genome reconstruction. Paired-end (PE) Illumina reads, at 25× coverage (350 ± 50-bp insert size), and 15-kb mate-pair (MP) reads (450 ± 50-bp insert size), obtained with a 454 Life Sciences FLX Titanium platform, were used to obtain a first draft. The remaining gaps were closed with 100× Illumina PE reads (6) and Sanger sequencing technology. The quality, accuracy, and misassemblies were checked with reference sequence-independent methods, i.e., feature response curve-based analysis (7), and the fragment coverage distribution method, using mapped PE and MP reads (8). Annotation was accomplished with the Prokaryotic Genome Annotation Pipeline (PGAP) of the NCBI (http://www.ncbi.nlm.nih.gov/genome/annotation_prok/).

The complete genome of *P. balearica* DSM 6083^T^ has 4,383,480 bp, an absence of extrachromosomal elements, and 64.7 mol% G+C content. Annotation revealed 4,042 predicted genes, including 3,923 open reading frames (ORFs) (97%). The majority of the putative protein-coding genes detected have significant matches with known or predicted functions (82.4%). Sixty tRNA genes representing all 20 amino acids are present, and 46 pseudogenes have also been detected. The presence and arrangement of 4 complete sets for rRNA genes on the assembled circular chromosome agree with previous results (9).

The carbohydrate metabolism capabilities detected include glycolysis, the tricarboxylic acid (TCA) cycle, the pentose phosphate pathway, and gluconeogenesis. The nitrogen cycle is represented by genes of denitrification, assimilatory, and dissimilatory nitrate reduction. Genes for flagellum synthesis and bacterial chemotaxis were found. The genome repertoire for bacterial secretion across the cytoplasmic membrane includes genes of the general secretory pathway type II, signal recognition particle, and Tat (twin-arginine translocation). However, no elements of type I, III (T3SS), V, or VII (chaperone/usher pathway) secretion systems were detected. A complete clustered regularly interspaced short palindromic repeat (CRISPR)-associated (Cas) system type I, subtype I-E (10), and a 54.9-kb size prophage were detected in the genome of *P. balearica* DSM 6083^T^.

A comparative genome sequence analysis based on average nucleotide identity based on BLAST (ANIb) values (11) clearly differentiated *P. balearica* DSM 6083^T^ from strains of the closest species (81.2% with *Pseudomonas stutzeri* ATCC 17588^T^).

### Nucleotide sequence accession number.

This complete genome sequence has been deposited in DDBJ/ENA/GenBank under the accession no. CP007511. The version described in this paper is the first version.
